# Mobility training using a bionic knee orthosis in patients in a post-stroke chronic state: a case series

**DOI:** 10.1186/1752-1947-6-216

**Published:** 2012-07-23

**Authors:** Nancy N Byl

**Affiliations:** 1 Department of Physical Therapy and Rehabilitation Science, School of Medicine, University of California San Francisco (UCSF), PT Health and Wellness Center, Bakar Community Center, 1675 Owens Street, Box 0736, San Francisco, 94158-2332, USA

## Abstract

**Introduction:**

An emerging area of neurorehabilitation is the use of robotic devices to enhance the efficiency and effectiveness of lower extremity physical therapy post-stroke. Many of the robotic devices currently available rely on computer-driven locomotive algorithms combined with partial bodyweight-supported treadmill training that drive reflex stepping with minimal patient intention during therapy. In this case series, we examined the effect of task-oriented mobility training in patients in a post-stroke chronic state using a novel, wearable, mobile, intention-based robotic leg orthosis.

**Case presentation:**

Three individuals, all of whom had reached a plateau with conventional bodyweight-supported treadmill training, participated in task-oriented mobility therapy (1.5 hours, two to four times per week for four weeks) with a robotic leg orthosis under supervision by a physical therapist. Participant 1 was a 59-year-old Caucasian man, who had an ischemic left stroke six years previously with resultant right hemiparesis. Participant 2 was a 42-year-old Caucasian woman with left hemiparesis after a right stroke 15 months previously. Participant 3 was a 62-year-old Caucasian woman with a history of a right middle cerebral artery aneurysm with third degree sub-arachnoid hemorrhage 10 years ago.

Immediately after training, all participants demonstrated improved gait speed (10 meter walk), stride length and walking endurance (6 minute walk) compared with baseline measurements. Improvements were maintained one month after training. Timed up and go and five times sit-to-stand were maintained for all three participants, with only one individual remaining outside the safety performance norm.

**Conclusions:**

Lower extremity training integrating an intention-based robotic leg orthosis may improve gait speed, endurance and community levels of participation in select patients in a post-stroke chronic state after plateauing within a bodyweight-supported treadmill training program. The wearable, mobile assistive robotic device safely supplemented supervised physical therapy including mobility and balance skill training.

## Introduction

Individuals who receive coordinated rehabilitation services after stroke demonstrate reduced mortality and achieve greater independence than those who do not [1]. Although early intervention and rehabilitation are preferred in the recovery process, research indicates late recovery may also occur for chronic stroke survivors. Late recovery mechanisms require patient effort and attention including progressive repetitive task-specific training to drive neural remodeling and reorganization [2].

An emerging area in neurorehabilitation is the use of robotic devices to enhance the efficiency and effectiveness of post-stroke therapy. A number of designs have been commercialized. These wearable, assistive robotic devices typically demand attention and repetition from the patient, while providing feedback through various modes to improve performance. Early studies of upper-limb robotic devices remain inconclusive when evaluating their benefit over conventional physical therapy [3]. Robotic systems for lower extremity therapy on a bodyweight-supported treadmill are subject to many of the same limitations as conventional bodyweight-supported treadmill training (BWSTT) [4,5] as they do not compensate for the lack of visual flow, balance and intention requirements inherent to this kind of movement training.

An analysis of the Framingham Study cohort found that as many as 30% of stroke survivors exhibit some kind of gait impairment [6], and after a stroke patients frequently demonstrate knee extensor weakness with decreased voluntary muscle activation [7,8]. Prior work suggests strength training can improve strength, gait speed and transitional movements in older adults [9-11] and using a robotic leg orthosis may enhance mobility [12]. However, the use of a mobile, intention-based robotic device designed to supplement knee extensor function in patients post stroke has not been investigated. In this three-case series, we examined the effect of task-oriented mobility training on gait speed, quality and endurance using a novel, mobile, intention-based robotic leg orthosis (RLO) after each individual, stable, chronic post stroke, had reached a plateau in gait performance following BWSTT [12].

## Case presentation

The specific aim of this study was to test the hypothesis that use of a RLO during a four-week training trial would improve mobility and balance in patients in a stable chronic state after a stroke who had reached a plateau with BWSTT (that is, cessation of training progress). The protocol was approved by the University of California, San Francisco, Committee on Human Research and participants gave written informed consent. Key eligibility and inclusion criteria included: 40 to 65 years of age; at least one year post stroke; the ability to walk at least 10 meters with or without an ankle-foot orthosis and/or cane; having reached a plateau in gait performance with BWSTT; and independence in self-care. Participants with medical instability, a major cardiopulmonary deficiency, major depression or significant cognitive deficit, or those currently receiving gait training were excluded. Table 1 summarizes baseline characteristics of the three participants, with additional narrative below.

**Table 1:** Bionic neurorehabilitation study: participant baseline characteristics

**Assessment value**	**Participant**		
	**1**	**2**	**3**
Sex	M	F	F
Age (years)	59	42	62
Ethnicity	Caucasian	Caucasian	Caucasian
Left lower extremity strength (lb)	169	90^a^	105^a^
Right lower extremity strength (lb)	97^a^	105	138
Resting extensor tone (Ashworth scale)^a^			
Ankle	2	4	1
Knee	4	3	1
Affected side	Right	Left	Left

Participant 1 was a 59-year-old Caucasian man, who had an ischemic left stroke six years previously with resultant right hemiparesis. He ambulated using a single-point cane and a hinged ankle-foot orthosis on his right leg. He used a hip-hike and circumduction strategy to clear his right foot during the swing phase of walking. During sit-to-stand activities he relied heavily on his uninvolved left side. He had eight weeks of BWSTT one month before the RLO study and was continuing general post-stroke therapy. During the RLO study he trained three times per week (11 visits).

Participant 2 was a 42-year-old Caucasian woman, with left hemiparesis after a right stroke 15 months previously. She ambulated using a wide-based quad cane and a hinged ankle-foot orthosis. She exhibited diminished weight acceptance on her involved left lower extremity and maintained excessive hip and knee flexion during a shortened stance phase of gait. She demonstrated a ‘step-to’ gait pattern. During sit-to-stand activities, she relied primarily on the use of her uninvolved right lower extremity. She completed BWSTT training one month before the RLO study and was continuing general therapy twice per week. During the RLO study she trained two times per week (eight visits).

Participant 3 was a 62-year-old Caucasian woman with a history of a right middle cerebral artery aneurysm with third degree sub-arachnoid hemorrhage 10 years ago. She had also undergone right knee replacement two years previously. She usually wore a hinged ankle-foot orthosis and used a single-point cane when ambulating in the community, but did not use her cane at home. She exhibited 10 to 15 degrees of genu recurvatum on her left lower extremity during the stance phase of gait. She also had difficulty with eccentric control during stand-to-sit and when descending on stairs. During sit-to-stand, she tended to adduct and internally rotate both hips, creating a valgus angle of her knees. She tended to stand with most of her weight on her less impaired right leg. She had completed BWSTT training several months prior to the RLO study. During the RLO study, she trained four times per week (15 visits).

**Figure 1:**
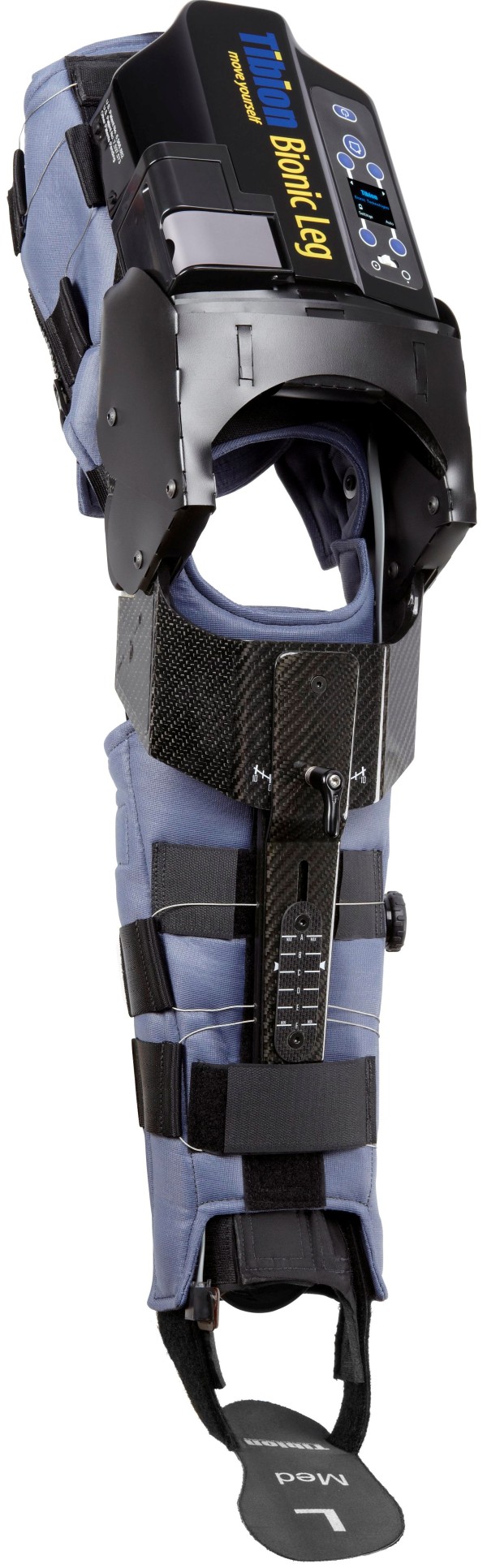
**Robotic leg orthosis. (a)** Tibion Bionic Leg orthosis and **(b)** shoe insert with foot sensor.

A portable, wearable, battery-powered RLO (Tibion Bionic Leg, Tibion Corporation, Sunnyvale, CA, USA) was used during therapy to actively supplement concentric and eccentric quadriceps function on the participants’ impaired side. The device uses internal sensors at the foot and knee joint to detect intention of movement and, once a variable force threshold is passed, it provides appropriate assistive and resistive adjustments (Figure 1). The RLO has three adjustable settings: threshold (the lower limit of force that must be crossed to initiate device assistance), assistance (the percentage of body weight provided through the limb during extension), and resistance (resistance to flexion during stair descent and stand-to-sit transfer). The therapist sets these parameters subjectively following evaluation, with the goal of balancing the physical contributions from the patient and the RLO during therapy.

The primary mode of operation is an automatic assist mode, in which the motorized actuator supplies forces that act against gravity based on the movement and biomechanics of the user. The device can provide assistance with extension (for stair ascent, stance phase, forward propulsion, and so on), controlled flexion (for stair descent or stand-to-sit) and free movement during the swing phase. The actuated RLO allows the user to engage his or her weaker leg more than would otherwise be possible, enabling the therapist to focus attention on patient midline positioning, symmetry and weight bearing through the involved lower extremity.

Participants received physical therapy using the RLO over a four-week period, at an individualized training frequency. Each training session was approximately 2 hours in length, with 1.5 hours allocated specifically for exercise. Programs were designed to require attention and repetition, and were similar for all participants, but adapted in difficulty to address the baseline ability of each participant. Each session began with a series of sit-to-stand transfers and standing squat activities. During walking, verbal and visual cues were given to focus the participant to actively use the involved limb, provide equal weight distribution through both lower extremities and attempt symmetrical steps emphasizing a ‘step through’ pattern. Depending on the participant’s ability, gait training was advanced to involve obstacle clearance, uneven terrain, community ambulation, forced speed on a treadmill, reciprocal stair climbing, sidestepping, backwards walking and walking without the use of a cane. During active therapy, the therapist provided stand-by guarding. Patients did not receive additional therapy in the one-month period following the RLO training.

The author assessed participants without the RLO at baseline, immediately following the completed course of therapy, and one month after therapy. Mobility assessments included the 10 meter walk test, 6 minute walk test and the Tinetti Gait Assessment [13]; balance assessments were the TUG test [14] and the FTSTS test [15]. Upon the completion of the study, all participants completed a questionnaire regarding their experience using the robotic device.

All three participants safely completed task-specific mobility practice with the RLO with minimal assistance from a physical therapist. Tables 1 and 2 summarize the baseline and post-intervention values for the functional outcome assessments measured in this case series. After four weeks of training with the RLO, each participant achieved measurable and clinically significant gains in gait speed (>0.1m/s) and endurance. Step length increased with training. Each participant reported feeling more stable and able to walk faster. Gains in gait speed and endurance were not consistent with the intensity of training. Two of the participants progressed in their gait classification - participant 2 from a household ambulator to a limited community ambulator and participant 1from a limited community ambulator to a community ambulator. Participant 3 began and ended the study as a community ambulator.

**Table 2:** Baseline and post-intervention values for functional outcome assessments*

**Test**	**Baseline**	**Post-training**	**Difference**^**a**^(%)*	**One month**	**Difference**^**a**^(%)*
**10 meter walk (m/s)**^a^					
Participant 1	0.60	0.81	35	0.95	58
Participant 2	0.29	0.43	48	0.45	55
Participant 3	0.83	1.03	24	1.14	37
**6 minute walk (m)**					
Participant 1	170	207	22	250	47
Participant 2	93	140	50	123	32
Participant 3	271	300	11	285	5
**Timed up and go (s)**^b^					
Participant 1	21.4	14.5	(32)	(12.4)	(42)
Participant 2	21.4	23.3	9	24.0	11
Participant 3	9.14	8.94	(2)	10.1	11
**Five times sit-to-stand (s)**^c^					
Participant 1	12.1	10.5	(13.7)	(11.1)	(8)
Participant 2	12.4	13.0	5	13.2	6
Participant 3	11.4	12.2	7	10.1	(11)
**Step length (m)**					
Participant 1	0.49	0.56	14	0.59	20
Participant 2	0.29	0.40	38	0.39	35
Participant 3	0.54	0.59	9	0.60	11

^*^Percentage change scores from baseline. Improvement is indicated by positive change scores for gait speed, endurance and step length and by negative change scores (indicated by parentheses) for five times sit-to-stand and timed up and go.

Normative Values Table 2 :

^a.^ Average walking speed for people 60 to 69 years of age averages 1.34 m/s for males and 1.24 m/s for females. Average self-selected walking speed for patients post stroke is 0.74 m/s; Ambulation can be further classified as :household ambulators, <0.4 m/s; limited ambulators, 0.4 to 0.8 m/s; unlimited community ambulators, >0.8m/s [13].

^b.^ Timed up and go > 13.4s indicates patients are at risk of falls [14].

^c.^ Five times sit-to-stand predicts falls when the time is >11.7s for patients 60 to 69 years; >12.6 s for patients 69 to 79 years; and >14.8 s for patients 80 to 89 years [15].

The gains were maintained or further improved at the one-month follow-up, in the absence of additional training. In a follow-up telephone contact approximately one year after the training, all three participants reported making continued improvements in community mobility and participation.

Changes in balance measures post training were less conclusive. At baseline, all participants performed within the age-expected normal values on the five times sit-to-stand test (FTSTS) (scores <14s). However, two of the participants exhibited slower than age-expected time on the timed up and go (TUG) test, both at the beginning and the end of the study.

## Discussion

When considering the clinical significance of the gait speed and endurance gains in this study, it is important to note the participants had reached a plateau from previous conventional BWSTT. Unlike BWSTT, the RLO employed in this study enabled patients to move in an untethered fashion, with movements that required a therapist-determined level of patient intention and balance during training. It is conceivable, although unlikely, that a learning effect could explain some of the gains. Gait practice with the RLO was multifactorial and included stair climbing, balance activities on unstable surfaces, ball kicking and other weight-shifting activities, treadmill walking and stepping up and down progressively high steps. The variability of practice activities facilitated by use of the RLO improves the likelihood that the post-therapy gains are attributable to differences in training approach. The persistence of the effect after the end of therapy, in spite of the previous plateau, further supports this interpretation, and is consistent with previous evidence that task-oriented training leads to the integration of functional gains into mobility strategies at home and in the community [16-19].

Lack of TUG improvement may have been due to the absence of specific training for quick directional changes and short-latency start and stop practice during walking. It is also possible that the tests used to measure balance were not sensitive enough to document changes in performance or confidence. Post-training knee extensor strength was not assessed.

No adverse events were noted during training. At the end of the training sessions, participants reported physical fatigue, attributable to the intensive physical activity during training. All participants reported positive experiences regarding their interaction with the RLO. Each indicated the RLO training made it easier for them to stand up, sit down and walk, not only during therapy but also at home and in the community.

## Conclusion

For patients who are stable after a stroke, this study investigated the benefit of intentional, task-oriented, untethered gait training with a novel, wearable, mobile RLO designed to assist knee function during transitional movements, walking, coordinated movements and stair climbing. The device was integrated into the rehabilitative process to actively assist hemiparetic patients to improve functional mobility and gait skills after they had plateaued with physical therapy including BWSTT. These results suggest additive clinical-functional benefits may be achieved by incorporating mobile, intention-based robotic technology into therapist-supervised mobility training for patients in the late-phase post stroke.

## Abbreviations

BWSTT, bodyweight-supported treadmill training; FTSTS, five times sit-to-stand; RLO, robotic leg orthosis; TUG, timed up and go.

## Consents

Written informed consent was obtained from the patients for publication of this case report and accompanying images. A copy of the written consent is available for review by the Editor-in-Chief of this journal.

## Competing interests

The author declares that she has no competing interests.
